# Electroencephalographic Measurement on Post-stroke Sensory Deficiency in Response to Non-painful Cold Stimulation

**DOI:** 10.3389/fnagi.2022.866272

**Published:** 2022-05-11

**Authors:** Yanhuan Huang, Jiao Jiao, Junyan Hu, Chihchia Hsing, Zhangqi Lai, Yang Yang, Zengyong Li, Xiaoling Hu

**Affiliations:** ^1^Department of Biomedical Engineering, The Hong Kong Polytechnic University, Hong Kong, Hong Kong SAR, China; ^2^Institute of Textiles and Clothing, The Hong Kong Polytechnic University, Hong Kong, Hong Kong SAR, China; ^3^Beijing Key Laboratory of Rehabilitation Technical Aids for Old-Age Disability, National Research Centre for Rehabilitation Technical Aids Beijing, Beijing, China; ^4^University Research Facility in Behavioral and Systems Neuroscience (UBSN), The Hong Kong Polytechnic University, Hong Kong, Hong Kong SAR, China; ^5^The Hong Kong Polytechnic University Shenzhen Research Institute, Shenzhen, China; ^6^Research Institute for Smart Ageing (RISA), The Hong Kong Polytechnic University, Hong Kong, Hong Kong SAR, China

**Keywords:** stroke, sensory deficiency, electroencephalography, elementary somatosensation, thermal sensation, non-painful cold stimulation

## Abstract

**Background:**

Reduced elementary somatosensation is common after stroke. However, the measurement of elementary sensation is frequently overlooked in traditional clinical assessments, and has not been evaluated objectively at the cortical level. This study designed a new configuration for the measurement of post-stroke elementary thermal sensation by non-painful cold stimulation (NPCS). The post-stroke cortical responses were then investigated during elementary NPCS on sensory deficiency *via* electroencephalography (EEG) when compared with unimpaired persons.

**Method:**

Twelve individuals with chronic stroke and fifteen unimpaired controls were recruited. A 64-channel EEG system was used to investigate the post-stroke cortical responses objectively during the NPCS. A subjective questionnaire of cold sensory intensity was also administered *via* a numeric visual analog scale (VAS). Three water samples with different temperatures (i.e., 25, 10, and 0°C) were applied to the skin surface of the ventral forearm for 3 s *via* glass beaker, with a randomized sequence on either the left or right forearm of a participant. EEG relative spectral power (RSP) and topography were used to evaluate the neural responses toward NPCS with respect to the independent factors of stimulation side and temperature.

**Results:**

For unimpaired controls, NPCS initiated significant RSP variations, mainly located in the theta band with the highest discriminative resolution on the different temperatures (*P* < 0.001). For stroke participants, the distribution of significant RSP spread across all EEG frequency bands and the temperature discrimination was lower than that observed in unimpaired participants (*P* < 0.05). EEG topography showed that the NPCS could activate extensive and bilateral sensory cortical areas after stroke. Significant group differences on RSP intensities were obtained in each EEG band (*P* < 0.05). Meanwhile, significant asymmetry cortical responses in RSP toward different upper limbs were observed during the NPCS in both unimpaired controls and participants with stroke (*P* < 0.05). No difference was found between the groups in the VAS ratings of the different temperatures (*P* > 0.05).

**Conclusion:**

The post-stroke cortical responses during NPCS on sensory deficiency were characterized by the wide distribution of representative RSP bands, lowered resolution toward different temperatures, and extensive activated sensory cortical areas.

## Introduction

Reduced somatosensation is common after stroke, with one in two stroke survivors experiencing this sensory deficit (Carey et al., 2018). It mainly impairs their discrimination in sensations of thermal, touch, and pain and further affects their ability of functional independence and overall quality of life (Carey, 1995; Kessner et al., 2016). Due to the learned non-use of the sensory affected limb, post-stroke motor restoration was reported to be hindered by the impaired somatosensation, which is typically represented by slower recovery, reduced movement control, and even lesser rehabilitation outcomes (Wu et al., 2006; De Diego et al., 2013; Serrada et al., 2019). Despite the high prevalence and apparent importance of somatosensation in post-stroke motor restoration, sensory deficiency and its functional evaluation received little attention, compared to those of the motor function (De Diego et al., 2013; Carey et al., 2016; Serrada et al., 2019).

To evaluate the sensory impairment, quantitative and precise measurement of somatosensory functions is crucial. It is known that the somatosensory functions could be divided into two subtypes, the elementary and the intermediate (Takeda et al., 2000; Satoh et al., 2002; Dijkerman and De Haan, 2007; Matsuda et al., 2020). The elementary somatosensory functions mainly consist of light touch, pain, thermal sensation, joint position sense, and vibration sense (Matsuda et al., 2020). The intermediate somatosensory functions are usually the integration of the multiple tactile related functions, including 2-point discrimination, tactile localization, weight, texture, and shape perceptions (Matsuda et al., 2020). The traditional clinical practices on post-stroke sensory evaluation were mainly focused on the intermediate somatosensation because it is generally believed that brain lesions, e.g., stroke, or brain trauma, mainly leads to the impairment of intermediate somatosensation (Head and Holmes, 1911; Takeda et al., 2000) rather than the elementary ones. For instance, the two-point discrimination (Dellon et al., 1987) and the Semmes-Weinstein monofilament tests (Bell-Krotoski and Tomancik, 1987) are two typical assessments widely used in clinical practice for multiple integrated sensory functions. Recently, evidence was found that brain lesions also led to disturbances of the elementary somatosensation. For example, infarction at the postcentral gyrus would cause disturbance of thermal sensation (Satoh et al., 2002). However, the measurement of elementary sensation is underdeveloped in traditional clinical sensory assessments. A challenge is the configuration of the measurement with minimum introduction of other unrelated sensory inputs, when performing an elementary somatosensory evaluation. In the current clinical measurements for stroke rehabilitation, both elementary and intermediate somatosensory functions, as well as the motor functions, are usually evaluated in a mixture related to daily tasks, e.g., the Fugl-Meyer Assessment (FMA) (Fugl-Meyer et al., 1974). Among them, somatosensory measurements are oversimplified in these mixed scales with limited resolution by subjective ordinal scales (Dellon et al., 1987). For example, although the Rivermead assessment of somatosensory performance contains the item on thermal sensation, it only has two grades of “warm” and “cold,” and requires the intact cognition of a subject to give subjective verbal response in the measurement as in most of the clinical somatosensory assessments (Steimann et al., 2012). Therefore, objective measurements with more precise control on the stimulation to evoke elementary somatosensory responses are preferred in the understanding of the related impairment after stroke.

The cortical responses to external sensory inputs could be objectively captured by brain imaging and electrophysiological technologies, e.g., functional magnetic resonance imaging (fMRI) and electroencephalography (EEG). fMRI has been applied in exploration of tactile, thermal, and pain stimulations to unimpaired persons with the advantage of its high spatial resolution (Chen et al., 2002; Mazzola et al., 2012). Relatively static spatial segregations in relation to these sensory inputs were observed (Chen et al., 2002; Mazzola et al., 2012). However, fMRI is hard to detect transient responses because of its lower temporal resolution compared to other modalities, e.g., EEG (Lystad and Pollard, 2009; Caliandro et al., 2017). EEG features represented by somatosensory evoked potential (SSEP) in time domain (Misra et al., 2008) and selected frequency components in EEG power spectra (Ahn et al., 2016) have been applied in objective measurement on cortical responses to sensory stimulations. SSEP parameters, e.g., onset value, peak intensity, duration, etc., have been widely used to determine the afferent neural integrity (Hwang et al., 2016; Yoon et al., 2018), based on the detection of the whole SSEP waveform with high repetitions to achieve a high signal-to-noise ratio (SNR) (Society, 2015). On the other hand, frequency features in the EEG power spectra, e.g., alpha and beta powers, could be less affected by the background noises to demonstrate the cortical responses to the sensory inputs, because of the less overlapped distribution between the selected power bands and those of the noises in the frequency domain (Thut and Miniussi, 2009). Therefore, EEG frequency features could be obtained with less repetitions, compared to those SSEP detections in the time domain (Thut and Miniussi, 2009). EEG spectral patterns also have been introduced to investigate the neural activities during the touch (Singh et al., 2014; Ahn et al., 2016) and thermal (Chang et al., 2005; Shao et al., 2012) stimulations in both unimpaired and stroke persons. Based on the study conducted by Singh et al. (2014), beta oscillation was found to be highly related to touch sensations, and its power changes could differentiate pleasant stimuli from unpleasant stimuli with different textile fabrics on a single trial basis. However, similar to the clinical manual assessments, the configurations in cortical measurements of elementary sensations were mixed with intermediate somatosensation in the reported EEG and fMRI studies (Kwan et al., 2000; Tseng et al., 2010; Mazzola et al., 2012; Ansari et al., 2018), e.g., the application of contact thermode with fastening belts for thermal stimulation to the skin would introduce multiple tactile and pressure stimuli, besides the thermal stimulation in the experiment (Kwan et al., 2000; Tseng et al., 2010).

In this study, we designed a new configuration for the measurement of post-stroke elementary thermal sensation by non-painful cold stimulation (NPCS), with minimized introduction of other sensations, such as tactile sensations with different textures and shapes. During thermal stimulation, pain sensation could be triggered and mixed with the thermal sensation according to an individual’s temperature threshold and the stimulus duration (Chang et al., 2005). Additionally, heat stimulation without pain (warm) is prone to make one drowsy, disturbing the cortical responses to the sensory inputs and lowering the efficiency of cortical network processing (Slater, 2008). Therefore, NPCS was selected to investigate the elementary thermal sensation in this study, with the purpose of minimizing the involvements of elementary pain sensation and potential disturbance from sleepiness. Then, we used quantitative EEG power spectra to investigate the cortical responses during the elementary NPCS on post-stroke sensory deficiency, when compared with unimpaired individuals.

## Methods

In this study, the post-stroke neural responses during the NPCS were investigated using EEG. A visual analog scale (VAS) questionnaire was administered to collect the subjective perceptions of cold stimulation. Three water samples, each at a different temperature, were applied to the forearm *via* glass beakers to induce the NPCSs in both stroke survivors and unimpaired individuals, with minimized disturbance of other sensory inputs. EEG power spectra and topography were used to evaluate the neural responses toward NPCSs.

### Participants

Before conducting the experiment, an approval was obtained from the Human Subjects Ethics Sub-Committee of the Hong Kong Polytechnic University. Twelve participants from local districts who entered the chronic stage after stroke were recruited to the “stroke group.” The following inclusion criteria were applied: (1) at least 6 months post-onset of a singular and unilateral brain lesion due to stroke; (2) the stroke-induced lesions occurred in the subcortical area; (3) an absence of visual, cognitive or attention deficits that would prevent participants from following instructions or performing the experimental procedures (assessed using the Mini-Mental State Examination (MMSE) score > 21) (Roselli et al., 2009); (4) presence of moderate-to-severe motor disability in the paretic limb [13 < Fugl-Meyer Assessment (FMA) < 47 (Fugl-Meyer et al., 1974; Woytowicz et al., 2016)] and muscle spasticity at the wrist and elbow [Modified Ashworth Score (MAS) ≤ 2 (Bohannon and Smith, 1987)]; (5) moderate-to-severe sensory impairment of their affected forearm, with a score of 1 as measured by the sensation part of light touch in FMA (Fugl-Meyer et al., 1974); (6) without symptom of central post-stroke pain (CPSP). The rationale for recruiting persons with chronic stroke was that their sensorimotor functions were relatively stable and were representative of a large population of stroke survivors. Meanwhile, to ensure the accessibility of cortical EEG responses, particularly those of the sensorimotor cortex, subjects with stroke and lesions in subcortical areas were recruited, since their cerebral cortex were not directly impaired due to stroke. Stroke patients with CPSP (overall prevalence around 6–8%; Krause et al., 2016) were excluded from this study to minimize bias, because patients with CPSP frequently show impairments of thermal and pain sensations that are different from patients without CPSP (Krause et al., 2016). The stroke group was subdivided into the “stroke affected group” and the “stroke unaffected group” according to the hemiplegic sides of the participants with stroke.

Fifteen unimpaired adults from local districts were recruited to the “control group.” They had no history of neurological, psychiatric, cognitive and/or cardiovascular diseases. All recruited participants gave written consent before the start of the experiment. The demographic characteristics of the participants are presented in Table 1. In this study, the recruited participants were mainly middle aged [45–65 years-old (Dictionary, 2016)], with a mean age of 55.13 years for the stroke group and 46.40 years for the control group. The difference in age was not statistically significant (*p* = 0.192, *t* = 1.341, independent *t*-test). However, significant difference in gender was found between the stroke participants and unimpaired controls (*p* = 0.005, Fisher’s exact test). In addition, all unimpaired participants and stroke survivors before their stroke onset were right-handed.

**TABLE 1:** Demographic characteristics of the participants.

Characteristics	Stroke group (*n* = 12)	Control group (*n* = 15)	*P*-values
Age in years^a^ (mean ± SD)	55.13 ± 16.04	46.40 ± 17.39	0.192
Gender^b^ (male/female)	11/1	5/10	0.005
Handedness before stroke^b^ (right/left)	12/0	15/0	1
Affected side (right/left)	6/6	Nil	Nil
Type of stroke (ischemic/hemorrhagic)	10/2	Nil	Nil
Times since stroke in years (mean ± SD)	14.92 ± 5.79	Nil	Nil
MAS elbow (mean ± SD)	1.08 ± 0.69	Nil	Nil
FMA full score for upper extremity (mean ± SD)	42.5 ± 15.17	Nil	Nil
FMA for light touch on forearm (mean ± SD)	1 ± 0	Nil	Nil

*^a^Independent t-test.*

*^b^Fisher’s exact test.*

### Electroencephalography Measurement of Cortical Response During Non-painful Cold Stimulation

#### Experimental Setup

**FIGURE 1:**
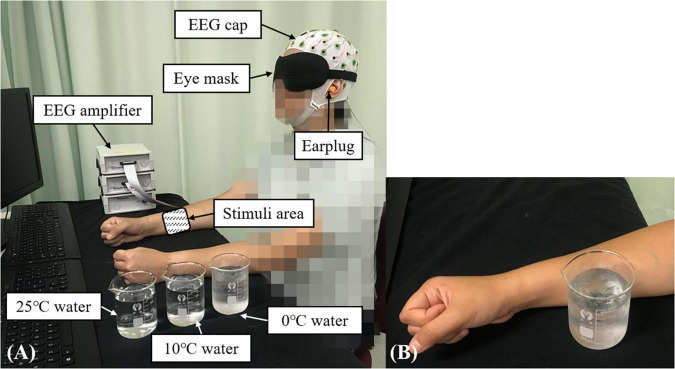
The experimental setup for the EEG evaluation during the non-painful cold stimulation (NPCS): **(A)** participant in the recording position; **(B)** demonstration of the NPCS.

The experiment was conducted in a lab which was controlled on temperature and humidity. The room temperature was maintained at 25 ± 1°C and the relative humidity was fixed at 60 ± 5%. Figure 1A shows the experimental setup. Each participant was comfortably seated in front of a table with both forearms placed on the table. Each participant wore an eye mask and ear plugs to minimize the disturbances from visual and audio stimuli from the surroundings. A 64-channel EEG system (BP-01830, Brain Products Inc.) was mounted on the scalp of each participant based on the standard 10–20 system, which was used to record the whole brain EEG with the skin impedance of each channel under 5 KΩ (Nuwer et al., 1999). The whole brain EEG recording was adopted because cold stimulation could activate multiple brain cortices in addition to the primary somatosensory cortex (Singh et al., 2014). The recording sampling frequency was set at 1,000 Hz.

#### Non-painful Cold Stimulation

In this study, the configuration for the measurement of post-stroke elementary thermal sensation using NPCS is shown in Figure 1B *via* glass beaker with different temperatures of water. During the stimulation, the glass beaker was designed to statically attach to the skin surface without friction, or load of weight. The selection of glass beaker was mainly due to its smooth surface and the monocomponent in the material, and these features could minimize the unnecessary sensory inputs of tactile sensations with different textures and shapes, when compared with the contact thermode fastened by belts used in the literature (Kwan et al., 2000; Tseng et al., 2010). The static attachment without friction and weight loading during the stimulation could further limit the introduction of friction sensation and weight perception. The NPCSs were delivered by three water samples with different temperatures of 25, 10, and 0°C, and the three temperatures could provoke weak to strong non-painful cold sensations, respectively. In the clinical application, 25°C usually acts as the adaptation temperature for control. The 10°C is frequently used for cool stimulation (Shao et al., 2012; Ansari et al., 2018), where the 0°**C** is a typical temperature for cold-induced pain. As mentioned above, pain sensation is related with the intensity and duration of the thermal stimulation (Chang et al., 2005). In unimpaired adults, exposing the skin to 0°C will evoke cold pain in 5 s (Rebbeck et al., 2015). To avoid the cold pain during the 0°C water stimulus, a 3-s short stimulation duration was used in this study. The target temperature was achieved by mixing room temperature water and different quantities of ice. The 0°C water was maintained as a mixture of ice and water during the whole experiment. Before applying each stimulus, a thermometer was used to ensure that the water temperature was within ±0.1°C of the target temperature. The NPCS with different temperatures was delivered to the skin surface of ventral forearm around the muscle belly of the *flexor carpi radialis* (FCR) and *flexor digitorum* (FD) muscles by the same experimenter. The FCR and FD muscles were selected because they were not only a common area to use in studies investigating the cold sensation for unimpaired participants (Chang et al., 2005), but also related to the wrist-hand flexion, which could promote post-stroke upper limb sensorimotor restoration for wrist-hand joints with appropriate stimulation (Lin et al., 2017). The estimated size of the contact area was around 3 cm × 3 cm.

#### Electroencephalography Measurement Protocol

**FIGURE 2:**
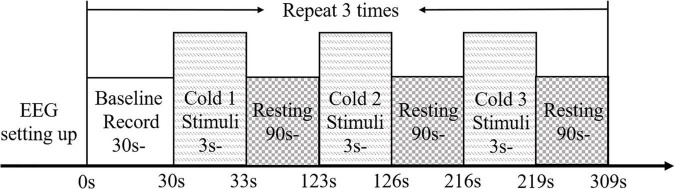
The experimental protocol for EEG evaluation presented with the timeline.

The timeline of EEG measurement during the NPCS is summarized in Figure 2. Each single trial of EEG measurement consisted of a 30-s baseline test, and three 3-s cold stimuli, which were separated by two 90-s resting times. Each participant was asked to keep their eyes closed, place both forearms on the table while remaining relaxed and still (Figure 1A). During the baseline test, no stimulation was applied to the participants, and they were required to remain awake without mental activity. During each NPCS, the glass beaker with a target temperature was statically attached to the skin surface around the FCR and FD muscles (Figure 1B), i.e., without friction, for 3 s. There was a computer program with timer to instruct the operator to deliver each NPCS. The operator would receive beep sounds though a headphone for instructions to start and stop. A countdown of the instruction would also be displayed on the computer screen as a reminder. Whether the stimulus was placed on the participant’s right or left forearm was determined by a randomized sequence. Participants were asked not to perform active cognition toward the cold stimuli during the EEG recording. The 90-s duration of rest was selected based upon the average clearance of cortical responses to cold stimulation in unimpaired persons (Garkavenko et al., 2008; Shao et al., 2012; De Pascalis et al., 2019). The cycles of EEG measurement for each participant were repeated three times on each forearm. The neural responses of both forearms were evaluated because the post-stroke cortical responses between different sides were varied during both sensory and motor tasks. This could be due to the different handedness before the stroke (Özcan et al., 2005; Dirnberger et al., 2011), and this asymmetric cortical response was also observed in our previous work in post-stroke fine touch to textile fabrics (Huang et al., 2020).

#### Electroencephalography Data Processing

After acquiring the targeted EEG data, the signals were processed off-line with a band-pass filter from 0.1 to 100 Hz, and a notch filter from 49 to 51 Hz to eliminate the 50 Hz noise from the environment. Following this, the EEG signals were divided into individual segments containing the baseline and cold stimulation periods. Then, the relative powers of each EEG frequency band, were calculated [i.e., Delta (δ, 0.1–4 Hz), Theta (θ, 4–8 Hz), Alpha (α, 8–13 Hz), Beta (β, 13–30 Hz), and Gamma (γ, 30–100 Hz) (Lebeau, 2010; Ahmed et al., 2012)]. The following equation was used:


(1) $${\text{P}}_{R\mathrm{elative}/b\mathrm{and}}=\frac{\int_{F_{1}}^{F_{2}}\text{p}\left(f\right)df}{\int_{0.1}^{100}\text{p}\left(f\right)df}-\frac{\int_{F_{1}}^{F_{2}}{\text{p}}_{B\mathrm{aseline}}\left(f\right)df}{\int_{0.1}^{100}{\text{p}}_{B\mathrm{aseline}}\left(f\right)df}$$

where, the P_*Relative/band*_ is the relative spectral power (RSP) of a frequency band; p(f) is the power spectral density of an EEG segment for a cold stimulation event, which is estimated by Fast Fourier Transform. F_1_ and F_2_ are the cutoff frequencies of a EEG frequency band, as stated above; and p_*Baseline*_(f) is the power spectral density of the EEG segments in the baseline tests of each trial. Similar analysis methods were adopted in our previous study (Huang et al., 2020).

### Non-painful Cold Sensation Evaluated by Questionnaire

The subjective evaluation of sensory intensities to the three cold stimuli were assessed using a numeric visual analog scale (VAS, range from 0 to 10), and it was conducted half an hour after the EEG measurement. Scores of 0–5 indicate no sensation to maximum non-painful cold sensation, while 6–10 score denotes minimum to maximum cold pain. During the VAS questionnaire, a non-painful cold stimulus was applied to the participants’ skin surface around the FCR and FD muscles, i.e., without friction, for 3 s. After removing the stimulus, participants were asked to rate the intensity of the cold sensation caused by the applied stimulus. The sequence for each cold stimulus was randomized. The subjective evaluation of cold sensations was also repeated three times on each forearm. Both forearms were assessed for the stroke participants, while only the dominant forearm (the right forearm in this study) was assessed for those unimpaired participants.

### Statistical Analysis

The normality tests for the EEG data and VAS scores were first evaluated using the Lilliefors method (Razali and Wah, 2011); the probabilities were statistically insignificant (*P* > 0.05), and the data obeyed normal distribution. During the intragroup comparisons, two-way repeated ANOVA was used to evaluate the RSP differences with respect to the independent factors of the stimulation side (i.e., left side and right side) and temperature (i.e., 25, 10, and 0°C) for each EEG frequency band. Then an independent *t*-test was used to evaluate the RSP differences with respect to the independent factor of stimulation side. One-way repeated measure ANOVA was conducted to investigate the RSP differences with respect to the independent factor of temperature with Bonferroni *post-hoc* test. During the intergroup comparisons, RSP differences of each EEG frequency band for each stimulation side with respect to the independent factors of the group and temperature were evaluated *via* mixed-model repeated measure ANOVA. Following this, one-way ANOVA was conducted to compare the group differences of RSPs on each stimulation side, with Bonferroni *post-hoc* test or Dunnett’s T3 *post-hoc* test. The selection of which *post-hoc* test to use was based on the homogeneity of the variance test. Specifically, for equal variance, the Bonferroni *post-hoc* test was adopted, otherwise, Dunnett’s T3 *post-hoc* test was used (Shingala and Rajyaguru, 2015). In addition, the intragroup VAS differences with respect to the independent factor of temperature were evaluated by one-way repeated measure ANOVA, while the intergroup differences of the VAS scores were calculated by one-way ANOVA, and both of them used Bonferroni *post-hoc* test. In this work, the level of statistical significance was set at 0.05, and the significance at levels 0.01 and 0.001 were also indicated.

## Results

**FIGURE 3:**
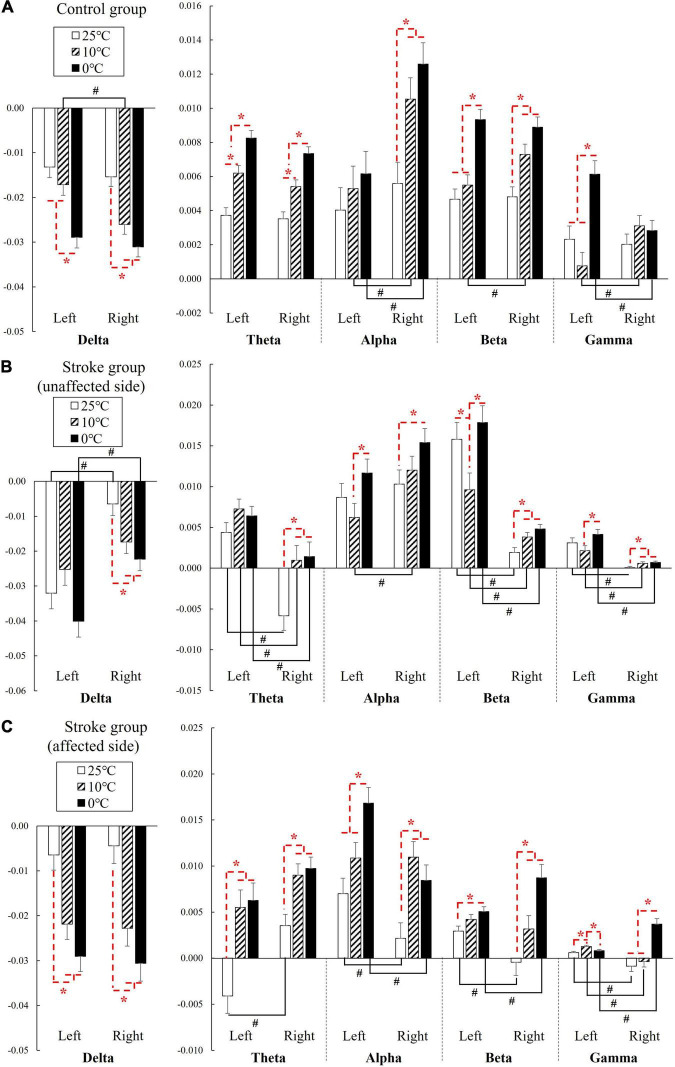
The EEG relative spectral power in response to the NPCSs on the left and right forearms for **(A)** control group, **(B)** stroke group-unaffected side, and **(C)** stroke group-affected side on the whole brain at the delta, theta, alpha, beta, and gamma band presented as mean value with SE (error bar). The significant intragroup differences are indicated by “*” (*p* < 0.05, one-way repeated ANOVA with Bonferroni *post-hoc* tests), and the significant intergroup differences are indicated by “#” (*p* < 0.05, independent *t*-test).

Figure 3 shows the EEG RSPs in response to the cold stimuli for each group at different EEG frequency bands. Table 2 presents the two-way repeated ANOVA probabilities and predicted effect sizes (EFs) with respect to the independent factors of the stimulation side and temperature. Table 3 provides a summary of the detailed values, means, and 95% CIs for each RSP, in addition to the one-way repeated ANOVA and independent *t*-test probabilities and EFs. In the control group (Figure 3A), significant RSP differences with respect to the temperatures were detected at all EEG frequency bands (*P* < 0.001, two-way repeated ANOVA, Table 2). In terms of the stimulation sides, significant differences were observed in the delta (*P* = 0.047, two-way repeated ANOVA, Table 2) and alpha (*P* = 0.001, two-way repeated ANOVA, Table 2) bands. Significant interactions between the temperature and the stimulation side were detected in the alpha (*P* = 0.042, two-way repeated ANOVA, Table 2) and gamma (*P* < 0.001, two-way repeated ANOVA, Table 2) bands. The theta RSPs of both sides in response to the 0°C water were significantly higher than those for 25 and 10°C stimuli (*P* < 0.001, one-way repeated ANOVA, Table 3), and the theta RSPs of both sides to 10°C water were significantly higher than those for 25°C water (*P* < 0.001, one-way repeated ANOVA, Table 3). The alpha and beta RSPs in response to 25°C were significantly lower than those in response to the 10 and 0°C water when the right side was stimulated (*P* < 0.001, one-way repeated ANOVA, Table 3). When the left side was stimulated, the beta and gamma RSPs in response to 0°C were found to be significantly higher than those at the 10 and 25°C water (*P* < 0.001, one-way repeated ANOVA, Table 3). Meanwhile, independent *t*-test established significant RSP differences in the delta, alpha, beta, and gamma bands for the different stimulation sides (*P* < 0.05, Table 3). The RSP of the right side was significantly lower than the left side in the delta band, while significantly higher in the alpha (*P* < 0.01, independent *t*-test, Table 3) and beta (*P* < 0.05, independent *t*-test, Table 3) bands. The gamma RSP for the right side was significantly higher than that for the left side when stimulated by the 10°C water (*P* = 0.012, independent *t*-test, Table 3), and significantly lower when stimulated by the 0°C water (*P* = 0.004, independent *t*-test, Table 3).

**TABLE 2:** Comparisons on the whole brain relative spectral power on each EEG frequency band.

		Two-way repeated ANOVA					
		Temperature		Stimulation side		Temperature × Stimulation side	
Bands	Groups	P (Partial η^2^)	F	P (Partial η^2^)	F	P (Partial η^2^)	F
Delta	Control group	<0.001^▲▲▲^ (0.030)	30.939	0.047^▲^ (0.004)	3.956	0.137 (0.002)	1.993
	Stroke group-affected side	<0.001^▲▲▲^ (0.062)	24.698	0.206 (0.004)	1.605	0.467 (0.002)	0.763
	Stroke group-unaffected side	<0.001^▲▲▲^ (0.033)	12.465	0.006^▲▲^ (0.020)	7.638	0.001^▲▲^ (0.020)	7.454
Theta	Control group	<0.001^▲▲▲^ (0.049)	50.654	0.058 (0.004)	3.595	0.593 (0.001)	0.523
	Stroke group-affected side	<0.001^▲▲▲^ (0.069)	27.437	0.019^▲^ (0.015)	5.547	0.395 (0.002)	0.929
	Stroke group-unaffected side	<0.001^▲▲▲^ (0.034)	13.102	0.255 (0.003)	1.301	0.097 (0.006)	2.336
Alpha	Control group	<0.001^▲▲▲^ (0.010)	10.323	0.001^▲▲^ (0.012)	11.554	0.042^▲^ (0.003)	3.178
	Stroke group-affected side	<0.001^▲▲▲^ (0.037)	14.302	0.490 (0.001)	0.477	0.232 (0.004)	1.463
	Stroke group-unaffected side	0.006^▲▲^ (0.014)	5.079	0.334 (0.003)	0.934	<0.001^▲▲▲^ (0.021)	7.938
Beta	Control group	<0.001^▲▲▲^ (0.033)	33.607	0.370 (0.001)	0.805	0.077 (0.003)	2.567
	Stroke group-affected side	<0.001^▲▲▲^ (0.034)	12.872	0.212 (0.004)	1.564	0.716 (0.001)	0.335
	Stroke group-unaffected side	<0.001^▲▲▲^ (0.037)	14.432	<0.001^▲▲▲^ (0.067)	26.648	<0.001^▲▲▲^ (0.020)	7.744
Gamma	Control group	<0.001^▲▲▲^ (0.010)	10.248	0.526 (0.000)	0.403	<0.001^▲▲▲^ (0.009)	9.436
	Stroke group-affected side	<0.001^▲▲▲^ (0.031)	11.943	0.002^▲▲^ (0.025)	9.379	0.099 (0.006)	2.319
	Stroke group-unaffected side	<0.001^▲▲▲^ (0.058)	23.024	<0.001^▲▲▲^ (0.034)	12.901	0.001^▲▲^ (0.018)	6.885

*Differences with statistical significance are marked with “▲” (p < 0.05, two-way repeated ANOVA intragroup tests on the temperature and stimulation side effects). Significant levels are indicated as, 1 superscript for < 0.05, 2 superscripts for < 0.01, 3 superscripts for < 0.001.*

**TABLE 3:** The whole brain relative spectral power of each non-painful cold stimuli for each group.

			25°C	10°C	0°C	One-way repeated ANOVA	
		
Bands	Groups	Stimulation side	Mean (95% confidence interval, E-03)			P (Partial η^2^)	F
Delta	Control group	Left forearm	–13.22 (–17.90 to –8.55)	–17.15 (–21.83 to –12.48)	–28.92 (–33.60 to –24.25)	<0.001*** (0.016)	16.457
		Right forearm	–15.37 (–19.73 to –11.02)	–26.04 (–30.39 to –21.69)	–31.08 (–35.43 to –26.73)	<0.001*** (0.017)	17.242
		Independent *t*-test P (Cohen’s d)	0.437 (0.036)	0.007^##^ (0.120)	0.554 (0.027)		
	Stroke group-affected side	Left forearm	–6.46 (–13.07 to –0.16)	–21.88 (–28.49 to –15.26)	–29.06 (–35.67 to –22.44)	<0.001*** (0.042)	16.459
		Right forearm	–4.42 (–12.24 to 3.41)	–22.78 (–30.61 to –14.95)	–30.60 (–38.43 to –22.77)	<0.001*** (0.042)	16.202
		Independent *t*-test P (Cohen’s d)	0.644 (0.035)	0.860 (0.013)	0.797 (0.018)		
	Stroke group-unaffected side	Left forearm	–32.00 (–40.87 to –23.14)	–25.27 (–34.13 to –16.40)	–40.10 (–48.97 to –31.24)	0.012* (0.012)	4.489
		Right forearm	–6.50 (–13.00 to 0.01)	–17.39 (–23.90 to –10.89)	–22.32 (–28.83 to –15.81)	<0.001*** (0.023)	8.728
		Independent *t*-test P (Cohen’s d)	<0.001^###^ (0.367)	0.147 (0.107)	0.004^##^ (0.209)		
Theta	Control group	Left forearm	3.72 (2.85–4.60)	6.20 (5.32–7.07)	8.25 (7.38–9.13)	<0.001*** (0.030)	30.250
		Right forearm	3.53 (2.74–4.31)	5.40 (4.61–6.19)	7.35 (6.56–8.13)	<0.001*** (0.025)	25.899
		Independent *t*-test P (Cohen’s d)	0.651 (0.021)	0.204 (0.057)	0.198 (0.064)		
	Stroke group-affected side	Left forearm	–4.10 (–7.80 to –0.39)	5.52 (1.82–9.22)	6.28 (2.57–9.98)	<0.001*** (0.045)	17.637
		Right forearm	3.54 (1.12–5.95)	9.01 (6.60–11.42)	9.75 (7.34–12.16)	<0.001*** (0.029)	11.133
		Independent *t*-test P (Cohen’s d)	<0.001^###^ (0.334)	0.137 (0.109)	0.188 (0.097)		
	Stroke group-unaffected side	Left forearm	4.39 (2.07–6.72)	7.27 (4.95–9.60)	6.40 (4.08–8.73)	0.119 (0.006)	2.144
		Right forearm	–5.82 (–9.37 to –2.28)	0.97 (–2.58 to 4.51)	1.41 (–2.13 to 4.96)	<0.001*** (0.036)	14.033
		Independent *t*-test P (Cohen’s d)	<0.001^###^ (0.401)	0.004^##^ (0.211)	0.038^#^ (0.153)		
Alpha	Control group	Left forearm	4.02 (1.43–6.62)	5.29 (2.69–7.88)	6.16 (3.56–8.75)	0.295 (0.001)	1.222
		Right forearm	5.58 (3.13–8.03)	10.53 (8.09–12.98)	12.59 (10.14–15.04)	<0.001*** (0.011)	11.449
		Independent *t*-test P (Cohen’s d)	0.320 (0.046)	0.006^##^ (0.123)	0.001^##^ (0.146)		
	Stroke group-affected side	Left forearm	7.00 (3.70–10.31)	10.88 (7.57–14.19)	16.84 (13.53–20.15)	<0.001*** (0.031)	11.694
		Right forearm	2.17 (–1.13 to 5.46)	10.96 (7.67–14.26)	8.44 (5.14–11.73)	<0.001*** (0.025)	9.568
		Independent *t*-test P (Cohen’s d)	0.017^#^ (0.174)	0.972 (0.003)	0.002^##^ (0.226)		
	Stroke group-unaffected side	Left forearm	8.69 (5.35–12.03)	6.22 (2.88–9.56)	11.67 (8.33–15.02)	0.034* (0.009)	3.433
		Right forearm	10.31 (6.93–13.70)	12.01 (8.62–15.39)	15.40 (12.01–18.78)	0.026* (0.010)	3.679
		Independent *t*-test P (Cohen’s d)	0.496 (0.049)	0.013^#^ (0.182)	0.146 (0.106)		
Beta	Control group	Left forearm	4.67 (3.48–5.85)	5.50 (4.32–6.68)	9.34 (8.15–10.52)	<0.001*** (0.020)	20.709
		Right forearm	4.81 (3.64–5.97)	7.30 (6.14–8.47)	8.90 (7.73–10.06)	<0.001*** (0.016)	16.625
		Independent *t*-test P (Cohen’s d)	0.851 (0.006)	0.028^#^ (0.099)	0.647 (0.019)		
	Stroke group-affected side	Left forearm	2.95 (1.92–3.98)	4.21 (3.19–5.24)	5.06 (4.03–6.08)	0.006** (0.013)	5.074
		Right forearm	–0.43 (–3.28 to 2.42)	3.18 (0.33–6.03)	8.72 (5.87–11.57)	<0.001*** (0.036)	14.015
		Independent *t*-test P (Cohen’s d)	0.013^#^ (0.184)	0.461 (0.052)	0.046^#^ (0.144)		
	Stroke group-unaffected side	Left forearm	15.81 (11.79–19.82)	9.62 (5.61–13.63)	17.86 (13.85–21.87)	<0.001*** (0.024)	9.215
		Right forearm	1.94 (0.79–3.08)	3.81 (2.67–4.96)	4.80 (3.66–5.95)	<0.001*** (0.021)	7.909
		Independent *t*-test P (Cohen’s d)	<0.001^###^ (0.482)	0.001^##^ (0.248)	<0.001^###^ (0.387)		
Gamma	Control group	Left forearm	2.32 (0.77–3.86)	0.77 (-0.78 to 2.31)	6.13 (4.59–7.68)	<0.001*** (0.014)	13.633
		Right forearm	2.03 (0.86–3.21)	3.11 (1.93–4.28)	2.83 (1.65–4.01)	0.286 (0.001)	1.251
		Independent *t*-test P (Cohen’s d)	0.752 (0.015)	0.012^#^ (0.111)	0.004^##^ (0.130)		
	Stroke group-affected side	Left forearm	0.60 (0.34–0.86)	1.27 (1.01–1.53)	0.81 (0.54–1.07)	0.001** (0.020)	7.695
		Right forearm	–0.86 (–2.02 to 0.31)	–0.37 (–1.53 to 0.80)	3.69 (2.52–4.85)	<0.001*** (0.054)	21.076
		Independent *t*-test P (Cohen’s d)	0.001^##^ (0.244)	0.001^##^ (0.264)	0.001^##^ (0.257)		
	Stroke group-unaffected side	Left forearm	3.11 (1.92–4.31)	2.16 (0.96–3.35)	4.16 (2.96–5.36)	0.004** (0.015)	5.701
		Right forearm	0.07 (–0.27 to 0.41)	0.61 (0.27–0.94)	0.71 (0.37–1.05)	0.003** (0.016)	5.978
		Independent *t*-test P (Cohen’s d)	<0.001^###^ (0.366)	0.006^##^ (0.207)	<0.001^###^ (0.353)		

Differences with statistical significance are marked with superscripts beside the P-values (“*” for one-way repeated ANOVA intragroup tests with Bonferroni post-hoc tests, “#” for independent t-test). Significant levels are indicated as, 1 superscript for < 0.05, 2 superscripts for < 0.01, and 3 superscripts for < 0.001.

In the stroke unaffected group (Figure 3B), two-way repeated ANOVA identified significant RSP differences with respect to the temperatures observed at all EEG frequency bands (*P* < 0.01, Table 2). Meanwhile, significant differences with respect to the stimulation sides were detected at the delta, beta, and gamma bands (*P* < 0.01, two-way repeated ANOVA, Table 2). In terms of the interaction between the temperature and the stimulation side, significances were found at the delta, alpha, beta, and gamma bands (*P* < 0.05, two-way repeated ANOVA, Table 2). One-way repeated ANOVA showed the RSPs of 25°C in the theta, beta, and gamma bands were significantly lower than that of 10 and 0°C for right-side stimulation (*P* < 0.01, Table 3). When the left side was stimulated, the significant variations between the RSPs of 10 and 0°C were observed in the alpha, beta, and gamma bands (*P* < 0.05, one-way repeated ANOVA, Table 3). Furthermore, significant differences were detected in all EEG frequency bands for the stroke unaffected group in respect of stimulation sides (*P* < 0.05, independent *t*-test, Table 3). Furthermore, the RSPs of the right side were significantly higher in the delta and alpha bands (*P* < 0.05, independent *t*-test, Table 3), and lower in theta, beta, and gamma bands (*P* < 0.05, independent *t*-test, Table 3), when compared with the left side.

In the stroke affected group (Figure 3C), significant differences with respect to the stimulation sides were detected at the theta (*P* = 0.019, two-way repeated ANOVA, Table 2) and gamma (*P* = 0.002, two-way repeated ANOVA, Table 2) bands. In terms of the temperature factor, significant differences were observed in all EEG frequency bands (*P* < 0.001, two-way repeated ANOVA, Table 2). No significant interactions between the temperature and the stimulation side was captured (*P* > 0.05, two-way repeated ANOVA, Table 2). One-way repeated ANOVA showed significant RSP variations in the delta (*P* < 0.001, one-way repeated ANOVA, Table 3), theta (*P* < 0.001, one-way repeated ANOVA, Table 3) and gamma (*P* = 0.001, one-way repeated ANOVA, Table 3) bands when the left side was stimulated with 25 and 10°C water. Additionally, significant RSP variations between the 25 and 0°C were found at the delta (*P* < 0.001, one-way repeated ANOVA, Table 3), theta (*P* < 0.001, one-way repeated ANOVA, Table 3), alpha (*P* < 0.001, one-way repeated ANOVA, Table 3), and beta (*P* = 0.006, one-way repeated ANOVA, Table 3) bands. When the right side was stimulated, the 25°C RSPs were significantly lower than those RSPs of 10 and 0°C in the theta, alpha, and beta bands (*P* < 0.001, one-way repeated ANOVA, Table 3). The gamma RSP for 0°C was significantly higher than that of both 25 and 10°C (*P* < 0.001, one-way repeated ANOVA, Table 3). Furthermore, there were significant RSP variations between the right side and the left side at theta, alpha, beta, and gamma bands, as determined by the independent *t*-test (*P* < 0.05, Table 3).

**FIGURE 4:**
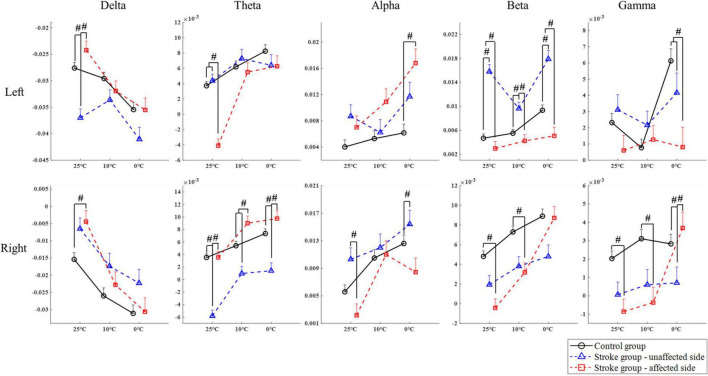
The EEG overall relative spectral power in response to the non-painful cold stimuli on the left and right forearms for each group at the delta, theta, alpha, beta, and gamma band, respectively, presented as mean value with SE (error bar). The significant intergroup differences are indicated by “#” (*P* < 0.05, one-way ANOVA with Bonferroni *post-hoc* tests or Dunnett’s T3 *post-hoc* tests).

Figure 4 compares the group differences of RSPs in response to the cold stimuli at each EEG frequency band with respect to the stimulation sides. Table 4 displays the values of statistical results including probabilities and EFs of the mixed-model repeated measure ANOVA and one-way ANOVA. Significant differences between both factors of groups and temperatures were found in the mixed-model repeated measure ANOVA for all the EEG frequency bands (*P* < 0.05, Table 4) on both stimulation sides. Significant interactions between the temperature factor and the group were recorded at delta and alpha band on the left side (*P* < 0.05, mixed-model repeated measure ANOVA, Table 4), as well as the theta, beta, and gamma bands on both sides (*P* < 0.05, mixed-model repeated measure ANOVA, Table 4). In comparison to the control group, when the left side was stimulated, the RSPs of the stroke affected group were significantly lower at the theta, beta, and gamma bands (*P* ≤ 0.001, one-way ANOVA, Table 4), and higher in the alpha band (*P* = 0.001, one-way ANOVA, Table 4). Meanwhile, when the right side was stimulated, the RSPs of the stroke affected group were significantly lower at the beta (*P* < 0.001, one-way ANOVA, Table 4) and gamma (*P* = 0.002, one-way ANOVA, Table 4) bands, and higher in the delta and theta bands (*P* < 0.001, one-way ANOVA, Table 4). Furthermore, significant RSP differences between the stroke affected group and the stroke unaffected group were noted in response to different temperatures across all frequency bands (*P* < 0.05, one-way ANOVA, Table 4). During the right-side stimulation, the alpha RSP of the stroke affected group was significantly lower than that of the unaffected group (*P* < 0.001, one-way ANOVA, Table 4), while the theta and gamma RSPs of the stroke affected group were significantly higher than those of the unaffected group (*P* < 0.05, one-way ANOVA, Table 4). During the left-side stimulation, the RSPs of the stroke affected group at theta, beta, and gamma bands were significantly lower than those of the unaffected group (*P* < 0.001, one-way ANOVA, Table 4), while the alpha RSPs of the stroke affected group were significantly higher than that of the unaffected group (*P* < 0.001, one-way ANOVA, Table 4). A comparison of the RSP values of the control group and the stroke unaffected group revealed significant differences in the theta, alpha, beta, and gamma bands (*P* ≤ 0.001, one-way ANOVA, Table 4) during the right-side stimulation. Similarly, significant differences were found in the delta, beta, and gamma bands (*P* < 0.05, one-way ANOVA, Table 4) during the left-side stimulation.

**TABLE 4:** Intergroup comparisons on the whole brain relative spectral power of each EEG frequency band.

			One-way ANOVA		Mixed-model repeated measure ANOVA					
	
			P (partial η^2^)	F	Temperature		Group		Temperature × Group	
Bands	Stimulation side	Temperature			P (partial η^2^)	F	P (partial η^2^)	F	P (partial η^2^)	F
Delta	Left forearm	25°C	<0.001^###^ (0.018)	15.943	<0.001^△ △ △^ (0.013)	22.970	<0.001^△ △ △^ (0.009)	7.724	0.017^△^ (0.003)	3.008
		10°C	0.165 (0.002)	1.801						
		0°C	0.090 (0.003)	2.414						
	Right forearm	25°C	0.003^##^ (0.007)	5.804	<0.001^△ △ △^ (0.021)	37.990	0.012^△^ (0.005)	4.456	0.315 (0.001)	1.186
		10°C	0.140 (0.002)	1.967						
		0°C	0.160 (0.002)	1.836						
Theta	Left forearm	25°C	<0.001^###^ (0.038)	34.097	<0.001^△ △ △^ (0.023)	40.855	0.002^△ △^ (0.007)	6.121	<0.001^△ △ △^ (0.009)	7.892
		10°C	0.587 (0.001)	0.532						
		0°C	0.328 (0.001)	1.117						
	Right forearm	25°C	<0.001^###^ (0.043)	38.554	<0.001^△ △ △^ (0.028)	50.435	<0.001^△ △ △^ (0.030)	26.779	0.003^△ △^ (0.005)	3.993
		10°C	<0.001^###^ (0.014)	12.355						
		0°C	<0.001^###^ (0.014)	11.969						
Alpha	Left forearm	25°C	0.052 (0.003)	2.961	<0.001^△ △ △^ (0.007)	11.405	0.001^△ △^ (0.008)	7.058	0.011^△^ (0.004)	3.260
		10°C	0.053 (0.003)	2.949						
		0°C	<0.001^###^ (0.011)	9.322						
	Right forearm	25°C	0.002^##^ (0.007)	6.088	<0.001^△ △ △^ (0.009)	15.911	0.022^△^ (0.004)	3.842	0.180 (0.002)	1.569
		10°C	0.818 (0.000)	0.200						
		0°C	0.051 (0.003)	2.985						
Beta	Left forearm	25°C	<0.001^###^ (0.044)	40.099	<0.001^△ △ △^ (0.011)	19.707	<0.001^△ △ △^ (0.039)	35.077	<0.001^△ △ △^ (0.008)	6.772
		10°C	<0.001^###^ (0.009)	7.685						
		0°C	<0.001^###^ (0.024)	20.947						
	Right forearm	25°C	<0.001^###^ (0.015)	12.796	<0.001^△ △ △^ (0.020)	35.387	<0.001^△ △ △^ (0.013)	11.568	0.002^△ △^ (0.005)	4.222
		10°C	<0.001^###^ (0.010)	8.586						
		0°C	0.009^##^ (0.005)	4.683						
Gamma	Left forearm	25°C	0.143 (0.002)	1.949	0.002^△ △^ (0.003)	6.022	0.013^△^ (0.005)	4.371	0.003^△ △^ (0.005)	3.985
		10°C	0.382 (0.001)	0.962						
		0°C	0.001^##^ (0.008)	7.010						
	Right forearm	25°C	<0.001^###^ (0.009)	7.791	<0.001^△ △ △^ (0.005)	8.678	0.001^△ △^ (0.008)	7.079	0.001^△ △^ (0.006)	4.817
		10°C	<0.001^###^ (0.009)	7.770						
		0°C	0.038^#^ (0.004)	3.288						

*Differences with statistical significance are marked with superscripts beside the P-values (“#” for one-way ANOVA intergroup tests with Bonferroni post-hoc tests or Dunnett’s T3 post-hoc tests, “△” for mixed-model repeated measure ANOVA intergroup tests on the temperature and group effects). Significant levels are indicated as, 1 superscript for < 0.05, 2 superscripts for < 0.01, 3 superscripts for < 0.001.*

**FIGURE 5:**
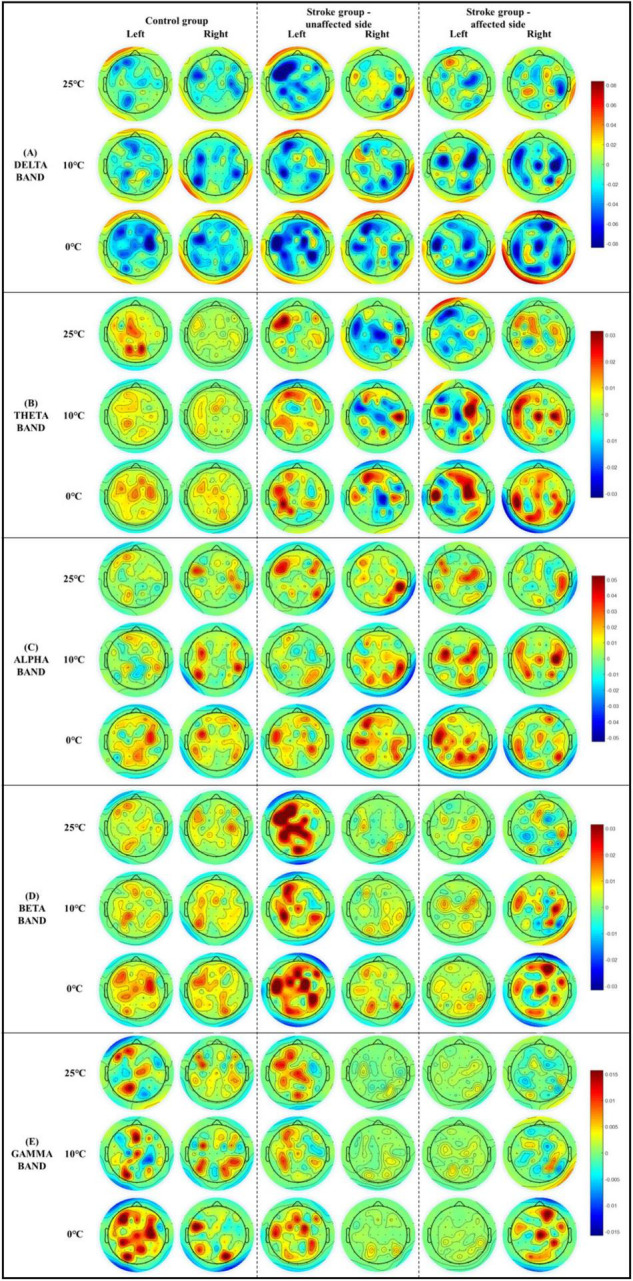
The whole brain EEG topography on the mean relative spectral power of **(A)** delta, **(B)** theta, **(C)** alpha, **(D)** beta, **(E)** gamma in response to the non-painful cold stimuli with respect to the stimulation side.

Figure 5 demonstrates the whole brain EEG topography of the mean RSPs in all EEG frequency bands for each group with respect to the independent factors of temperature and stimulation side. Hotspots of significant RSPs in all EEG frequency bands were captured bilaterally, mainly in the parietal, frontal, and occipital regions of both stroke and unimpaired participants. In the control group, there were increased theta power (Figure 5B) in the parietal and occipital regions bilaterally. The powers of high frequency bands (i.e., beta and gamma bands, Figures 5D,E) were increased not only in the parietal and occipital regions, but also in the occipital lobe. For stroke participants who had experienced right-side brain lesions (left hemiplegia), theta activity was increased in the bilateral parietal areas and decreased in the paramedian central area for both stimulation sides. Brain activity in the beta band (Figure 5D) only slightly increased in the bilateral parietal regions for both stimulation sides. In contrast, for participants with stroke who had experienced left-side brain lesions (right hemiplegia), theta activity (Figure 5B) increased in the left hemisphere of the brain when the unaffected left forearms were stimulated, while a remarkable increase in theta activity could be observed over the frontal, parietal, and occipital lobes when their affected right forearms were stimulated. In terms of the high frequency bands (i.e., beta and gamma bands, Figures 5D,E), brain activity in the central areas were significantly increased for the affected right forearms, where the 0°C stimulus initiated greater brain activity than the 25 and 10°C stimuli. Meanwhile, high frequency brain activities increased significantly almost over the whole brain when the unaffected left forearms were stimulated.

Table 5 presents the means and 95% CIs of each NPCS evaluated by VAS, in addition to the statistical probabilities and the EFs. The results demonstrated that the three temperatures of cold water could provoke weak to strong non-painful cold sensations in both stroke and unimpaired participants. Significant VAS variations with respect to the different temperatures were observed for each group *via* one-way repeated ANOVA (*P* < 0.001, Table 5). No statistical difference was found in the intergroup comparison for each cold stimulus (*P* > 0.05, one-way ANOVA, Table 5).

**TABLE 5:** Non-painful cold sensation evaluated by visual analog scale.

Groups	25°C	10°C	0°C	One-way repeated ANOVA	
		
	Mean (95% confidence interval)			P (partial η^2^)	F
Control group	1.40 (1.12–1.68)	3.27 (2.99–3.55)	4.60 (4.32–4.88)	<0.001*** (0.888)	110.480
Stroke group-unaffected side	1.42 (1.09–1.74)	3.25 (2.92–3.58)	4.67 (4.17–4.83)	<0.001*** (0.893)	91.432
Stroke group-affected side	1.50 (1.14–1.86)	3.67 (3.30–4.03)	4.92 (4.55–5.28)	<0.001*** (0.920)	125.673
**One-way ANOVA**					
P (Partial η^2^)	0.871 (0.008)	0.181 (0.091)	0.180 (0.091)		
F	0.138	1.795	1.797		

Differences with statistical significance are marked with “*” beside the P-values (one-way repeated ANOVA intragroup tests with Bonferroni post-hoc tests). Significant levels are indicated as, 1 superscript for < 0.05, 2 superscripts for < 0.01, 3 superscripts for < 0.001.

## Discussion

This study designed a novel configuration for the measurement of post-stroke elementary thermal sensation with NPCS. We utilized glass beaker with different temperatures of water, which statically attached to the skin surface without friction and load, to deliver the NPCSs with different intensities. In the literature, cold stimuli were mainly delivered by thermal pack (Mazzola et al., 2012; Ansari et al., 2018), thermode (Kwan et al., 2000; Tseng et al., 2010), or direct contact with the water (Maihöfner et al., 2002; Fardo et al., 2017), and these approaches unavoidably introduced mixed sensory inputs other than the thermal sensation. For example, direct contact with the water would mix the thermal sensation and the wetness sensation, while thermal pack and thermode with fasteners would introduce the intermediate texture, shape, or even weight perceptions. In comparison with those configurations in the literature, the involvements of other unrelated sensory inputs like texture, shape, wetness, and weight perceptions were effectively minimized with the configuration of this study. Meanwhile, the pain sensation was also avoided in the configuration with the precise 3 s stimulus duration. The NPCSs of this study were further verified by the VAS questionnaire (Table 5), where the VAS scores of all stroke and unimpaired participants were lower than 6 indicating without cold pain.

We measured the post-stroke sensory deficiency on the elementary non-painful cold sensation using both EEG RSP and VAS questionnaire. The VAS scores indicated that both stroke and unimpaired participants could distinguish the different NPCSs with statistical significance (Table 5). However, there was no significant intergroup difference in VAS scores between the stroke and unimpaired participants (Table 5), which could not further assess the post-stroke sensory alteration on thermal sensation. The insignificant intergroup VAS result could be due to the compensation of voluntary cognition toward sensory inputs for those stroke participants, leading to high thermal discrimination (Barulli and Stern, 2013). During the VAS questionnaire, participants were asked to rate the cold sensation provoked by each NPCS. This cognitive process could be related to the individual experiences, and mainly consisted of four primary elements including comprehension, retrieval, judgment, and response (Sudman et al., 1997; Tourangeau et al., 2000). On the contrary, significant group differences in cortical responses were found between the stroke and unimpaired groups toward different NPCSs. That is because the cortical responses captured by EEG RSPs were associated with involuntary attention drawn by the NPCS. During the EEG recording, to minimize the bias caused by the participants’ active cognitive process, the participants were requested to remain awake yet mentally inactive. Therefore, the EEG RSP could reveal much more preliminary cortical response on the post-stroke sensory deficiency than the compensated cognitive process during the VAS questionnaire rating.

The EEG results of the unimpaired participants (Figure 3A) highlighted that different NPCSs could induce significant RSP variations across the target frequency bands (i.e., theta, alpha, and beta bands). These three bands were activated with increased power during the NPCS. In line with our observations of enhanced EEG beta RSP in both fronto-temporal and parietal brain regions (Figure 5D), it has been reported in the literature that beta activity increased bilaterally in the fronto-temporal cortex in unimpaired people during the cold stimulation, especially during the cold pain (Chang et al., 2002; De Pascalis et al., 2019). Chang et al. (2002) suggested that the increased beta power was related to the frontal and temporal muscle activities in response to cold stimulation, caused by situational tension factors like stress or pain. The increased beta activities were then suspected to associate with residual muscle artifact, as EEG artifact. However, the increased beta power of this study was mainly located in sensorimotor cortex (Figure 5D), not closely to the frontal or temporal muscles, which could not be accounted as muscle artifact. In addition, the present results also found that beta RSP intensities were increased with the increase of stimulus intensities. De Pascalis et al. (2019) reported a similar observation during the cold pain, and they found that the higher EEG beta power in the left frontal, midline central, posterior temporal cortices were correlated with the higher cold pain scores (De Pascalis et al., 2019). Together with the increased beta power during the NPCS in this work, it implied that the beta power could be proportional to the intensities of cold stimulations whether it was painful or non-painful. It was also observed that both theta and alpha RSPs were increased during the NPCS. It could be the involuntary attention aroused during the non-painful thermal stimulation, i.e., outside events attract spontaneous attentional processes (Eimer et al., 1996; Iannetti et al., 2008; Wang et al., 2010), for example, to process the different NPCSs in this work. Chen et al. (2017) reported that the intensity of attentive oscillation (i.e., theta and alpha bands) increased with the difficulty of sensory tasks. The attentive oscillation would be relatively low when processing an easy task, while its intensity would be higher when a difficult task lasts for a longer duration to obtain more information (Chen et al., 2017). These are consistent with our findings (Figure 3A), among the three stimuli, the 0°C stimulus obtained the highest RSP intensity, and the RSP of 10°C was higher than that of the 25°C stimulus. Of the three representative frequency bands, the theta band showed the highest sensitivity in distinguishing different cold stimuli in unimpaired participants, because theta RSP obtained statistical differences between three cold stimuli for both stimulation sides. The limited sensitivity toward cold stimuli in the alpha and beta RSPs were possibly due to the dominance of the theta band during involuntary attention evoked by the sensory stimulation, as designed in this work.

Post-stroke neural responses of the elementary cold sensation induced by NPCS were also investigated for both affected and unaffected sides in the stroke participants. In the stroke unaffected group, the sensitive EEG RSPs of distinguishing different cold stimuli were concentrated in the beta and gamma bands (Figure 3B). Varied with the control group, the theta band was no longer the dominant frequency band toward cold stimuli in the stroke unaffected group, and it only remained limited resolution to different temperatures during the right-side stimulation. Instead, significant variations were observed in beta and gamma RSPs during the NPCS on both stimulation sides, whereas its thermal resolution was also limited. For example, both beta and gamma RSPs of the right side could not distinguish the difference between the 10°C and the 0°C water. The sensory deficiency in the unaffected upper limb of stroke survivors was frequently overlooked in routine stroke clinical practice. Recently, it has been pointed out that sensorimotor deficits frequently occurring in the unaffected upper limb were mainly due to the transcallosal interactions between the hemispheres (Grefkes and Fink, 2011), and decreased sensory discrimination is one of the representative symptoms for sensory deficiency in the unaffected upper limb. In this study, the reduced sensory discrimination toward elementary cold sensation for the unaffected side of the stroke participants was captured.

For the stroke affected group, in comparison with both unimpaired participants and the unaffected side of stroke survivors, a wider distribution of representative RSP across the whole spectrum toward NPCSs was observed, and all EEG frequency bands showed significant RSP differences toward the different cold stimuli (Figure 3C). These broadly activated EEG frequency responses could be related to the excessive cortical effort after stroke. It indicated that the desired sensory responses were challenging for chronic stroke survivors, even after the recovery period of pathological reorganization (Thibaut et al., 2017). It was widely reported that excessive cortical effort was required in post-stroke motor recovery when a task became more complex (Jones and Adkins, 2015; Thibaut et al., 2017). In this work, we observed the similar additional cortical effort in the sensory experiences during the NPCS. Meanwhile, although significant RSP variations toward different cold stimuli could be obtained in EEG frequency bands for the affected sides, their thermal resolutions on different temperatures represented by EEG RSP were low. For example, in unimpaired participants, the theta RSP demonstrated the highest sensitivity in distinguishing the three cold stimuli on both sides, while the theta RSP did not show a significant difference between the 10°C and the 0°C water on both sides for the stroke affected group. Another finding related to the power spectrum was the involvement of high frequency gamma band during the NPCS to the affected limbs of the stroke participants (Figure 3C). It could be due to the compensation of the impaired afferent proprioception and efferent control in sensorimotor system (Chen et al., 2018). Increased unconscious employments of higher-level attention and behavioral processes were usually observed in stroke survivors, when compared with the unimpaired individuals (Bannister et al., 2015; Chen et al., 2018). Previous studies also highlighted the contributions of post-stroke gamma activation, i.e., contributed to cognitive motor tasks (Gross and Gotman, 1999; Meador et al., 2002), and acted as a coding feature for functional prevalence in hand sensory areas (Tecchio et al., 2003, 2004). In our previous studies on post-stroke sensory deficiency during mechanical tactile stimulation (i.e., textile fabric stimulations), similar engagements of high frequency bands (i.e., beta and gamma bands) were observed in persons with chronic stroke (Huang et al., 2020). It further indicated the importance of gamma oscillation for somatosensory functions, including thermal and tactile sensations, following a stroke.

We also investigated the variations of neural responses during the NPCS between the left and right sides for each group. These variations were mainly investigated from two aspects, which included the RSP intensity and its resolution. From the resolution aspect, the RSP resolutions of the control and stroke affected group toward different cold stimuli between the left and right sides were comparable (Figures 3A,C). From the intensity aspect, the control group obtained significantly higher RSP intensities in the relative high frequency bands (i.e., alpha, beta, and gamma bands) during the right-side stimulation than left-side stimulation (Figure 3A). This could be related to the higher amplitude of cortical potentials during the dominant hand movement, in comparison with the non-dominant hand (Tarkka and Hallett, 1990). In this study, all the unimpaired participants were right-handed. In the stroke affected group, the right-side stimulation achieved significant higher RSP intensities in the relative high frequency bands (i.e., alpha, beta, and gamma bands) during the strong NPCS (i.e., 0°C) than the left-side stimulation, while the right-side stimulation obtained significant lower RSP intensities during the weak NPCS (i.e., 25°C) (Figure 3C). It might imply that the affected left forearm is more sensitive to a weak stimulus, where the affected right forearm is more sensitive to a high stimulus. This particular finding could be related to the lateralization of brain function, which result in the differences of behavior and recovery outcome between the left hemiplegia and right hemiplegia (Chen et al., 2000; Park and Lee, 2011; Frenkel-Toledo et al., 2019). For example, patients with left-hemisphere stroke often develop a slow and cautious behavioral style; on the contrary, those patients with right-hemisphere stroke usually present a quick and overly curious behavior (Hayn and Fisher, 1997). Owing to the superiority of controlling complex motor movements in the left hemisphere (Serrien and Spapé, 2009), it was also reported that patients with right hemiplegia had slower motor restoration in gait functions (Laufer et al., 2003; Gama et al., 2017). However, few works have been done on the differences in sensory impairment after the left- and right-hemisphere lesions, which necessitate further investigations (Goodin et al., 2018).

When it comes to the stroke unaffected group, asymmetric neural responses from both aspects were observed in all EEG frequency bands, especially in the high frequency bands (i.e., beta and gamma bands, Figures 3B, 5). Firstly, the thermal resolution of different temperatures for right unaffected forearms was higher than those of the left. In the theta band, significant RSP variation toward different cold stimuli was only observed during the right-side stimulation. In the gamma band, the RSP of the right forearm could significantly distinguish the 25°C stimulus from the other two stimuli, where the RSP of the left forearm could not. Secondly, the RSP intensities (theta, beta, and gamma bands) were significantly higher for the left unaffected forearms than the right ones (Figures 3B, 5). This excessive neural response suggested that additional cortical efforts were needed during the neural processing of thermal inputs for the unaffected left forearms. The imbalance of neural responses toward thermal stimulation for the stroke unaffected group could be related to the handedness alteration after stroke. Typically, dominant hands have better sensorimotor performance during single-handed tasks, like better manual dexterity during motor movements, and higher sensation thresholds for touch and pain than non-dominant hands (Özcan et al., 2005; Dirnberger et al., 2011). Most stroke survivors will use their unaffected forearms to perform sensorimotor tasks in their daily lives after stroke. In some stroke survivors with affected right dominant forearms, the unaffected left forearms will gradually become their new dominant upper limbs (Harris and Eng, 2006). However, it can be difficult for stroke survivors during the handedness alteration, with the unaffected left forearms presenting low sensation resolution and poor control over voluntary motor tasks, compared to their original dominant right arms. In this study, all stroke survivors were right-handed before their stroke onset. Among them, half were right hemiplegics, who had experienced handedness alteration after stroke. The RSP results of this study further verified the thermal sensitivity during the NPCS for the unaffected left forearm was lower than the unaffected right ones. In line with our previous study (Huang et al., 2020), the unaffected right forearms also showed better touch discrimination to the different fabrics during the tactile stimulation when compared with the unaffected left forearms.

Significant intergroup differences of RSP intensities were also observed (Figure 4). Significant variation on RSP intensity between the stroke unaffected group and control group was mainly captured in the beta band (Figure 4). When the left sides were stimulated, the intensity of beta RSP in the unaffected group was significantly higher than that in the control group. This might also be related to the alteration in handedness after stroke. When compared with the control group, the stroke affected group displayed sensory deficiency related to attenuated cortical responses during the weak cold stimuli (i.e., 10 and 25°C). When the right side was stimulated with the 10 and 25°C water, the RSP intensities of high frequency bands (i.e., beta and gamma bands, Figure 4) in the stroke affected group were significantly lower than the control group; however, the RSP intensities between the two groups were comparable when stimulated by 0°C. The cortical activation in the stroke affected group was relatively low during the processing of weak to moderate NPCSs (i.e., 10 and 25°C), while strong NPCS (i.e., 0°C stimulus) could initiate high cortical activation. It implied that the reduced somatosensation after stroke was mainly characterized as the insensitivity to those weak sensory inputs and their discriminations.

The sensory cortical centers in response to elementary thermal stimulation for both stroke and unimpaired participants were revealed by the whole-brain RSP topography (Figure 5). For those unimpaired participants, multiple brain regions were activated bilaterally during the NPCS, which included the primary sensorimotor cortex, insula cortex, occipital cortex, prefrontal, and frontal cortices. These findings were in line with the literature (Chang et al., 2002, 2005; De Pascalis et al., 2019), and further verified the cortical processing of thermal stimulation was a distributed system, rather than a strict somatotopic arrangement (Berman et al., 1998; Chang et al., 2005). In several fMRI studies, innocuous thermal-related activations in the anterior cingulate cortex and lateral-thalamus pathway were also reported (Davis et al., 1998; Kwan et al., 2000; Mazzola et al., 2012). For stroke survivors, the brain cortices that process non-painful cold sensation were also activated bilaterally in multiple brain regions as seen in unimpaired individuals, where the activated regions were more extensive with higher intensity. Similar distribution of post-stroke cortical activation after thermal stimulation was reported by one fMRI study (Chen et al., 2020). They observed that brain activations promoted by thermal stimuli were in the inferior-middle frontal cortex, superior-middle temporal cortex, insula, and primary sensorimotor cortex (Chen et al., 2020).

One of the concerns in the EEG measurements is the high signal to noise ratio (SNR) to ensure the signal quality, and it could be achieved by ensemble mean *via* repetitions (Väisänen and Malmivuo, 2009). In this study, we preferred EEG power spectra to investigate the cortical responses to NPCS, since it requires less repetition or signal length than the measurement of intact waveform of SSEP in the time domain. It is because the frequency components are less affected by the background noises, e.g., environmental noise (50 Hz power line) and spontaneous EEG unrelated to the stimulation, as the target EEG frequency features may not be overlapped with the noise features in the spectrum (Thut and Miniussi, 2009). For instance, the SSEP waveform detection usually requires averaging over 250–1,000 repetitions to obtain a reasonable SNR (Society, 2015), while Singh et al. (2014) only used a single trial to achieve the significant power changes between the pleasant and unpleasant touch stimuli.

The small sample size was one of the limitations of this study. However, under the current small sample size, significant differences in EEG RSP between the unimpaired population and the chronic stroke population had been obtained. Another limitation of this study was the significant gender differences between the two groups (*p* = 0.005, Fisher exact test, Table 1). In this study, we focused on the investigation of the impairment in elementary thermal sensation introduced by the neurological lesion after stroke, rather than the difference of gender. Gender differences in post-stroke sensory impairment, e.g., thermal sensation, has not been investigated yet. In future work, more stroke participants in different recovery stages, gender, age groups, and lesion sites will be investigated for a more comprehensive understanding.

## Conclusion

The contribution of this study was the objective and quantitative evaluation of post-stroke elementary thermal impairment toward non-painful cold stimulation *via* EEG RSP analysis, with a new configuration minimizing the unnecessary sensory inputs. The characteristics of cortical responses during the NPCS following a stroke were (1) wide distribution of representative RSP bands, (2) limited sensitivity toward different thermal stimuli, (3) more extensive sensory cortical areas, when compared with the EEG RSP features of the unimpaired control. Meanwhile, the post-stroke somatosensation of unaffected side was also impaired, which was represented by lower resolution toward different NPCSs and asymmetric RSP intensities toward different stimulation sides. EEG RSP pattern could quantify the neural responses of elementary thermal sensation after stroke, and enhance the understanding of post-stroke sensory deficiency on elementary thermal sensation.

## Data Availability Statement

The datasets presented in this article are not readily available because it has been stated in the consent approved by the Human Subjects Ethics Sub-Committee of the Hong Kong Polytechnic University that the results of the experiment may be published, but the individual results should be kept confidentially for each subject. Requests to access the datasets should be directed to XLH, xiaoling.hu@polyu.edu.hk.

## Ethics Statement

The studies involving human participants were reviewed and approved by the Human Subjects Ethics Sub-Committee of the Hong Kong Polytechnic University. The patients/participants provided their written informed consent to participate in this study.

## Author Contributions

YHH contributed to the experimental design, data analysis, and manuscript drafting. JYH and ZYL contributed to the experimental design. JJ, CCH, ZQL, and YY contributed to the subject management and experimental process. XLH conceived of the study and coordinated the whole project, including the study design, human subject experiments, and manuscript revising. All authors read and approved the final manuscript.

## Conflict of Interest

The authors declare that the research was conducted in the absence of any commercial or financial relationships that could be construed as a potential conflict of interest.

## Publisher’s Note

All claims expressed in this article are solely those of the authors and do not necessarily represent those of their affiliated organizations, or those of the publisher, the editors and the reviewers. Any product that may be evaluated in this article, or claim that may be made by its manufacturer, is not guaranteed or endorsed by the publisher.
